# Maternal immunoglobulin treatment can reduce severity of fetal acetylcholine receptor antibody-associated disorders (FARAD)

**DOI:** 10.1186/s42466-023-00280-6

**Published:** 2023-10-26

**Authors:** Matthias Wassenberg, Andreas Hahn, Anna Mück, Heidrun H. Krämer

**Affiliations:** 1 Department of Neurology, Justus Liebig University, Giessen, Germany; 2 Department of Child Neurology, Justus Liebig University, Giessen, Germany

**Keywords:** Fetal acetylcholine receptor inactivation syndrome, Fetal acetylcholine receptor antibody-associated disorders, Myasthenia gravis, Neuromuscular disorders

## Abstract

**Background:**

Fetal acetylcholine receptor antibody-associated disorders (FARAD), caused by in utero exposure to maternal antibodies directed against the fetal acetylcholine receptor (AChR), is a rare condition occurring in newborns of myasthenic mothers. Only two cases of FARAD children born to asymptomatic mothers are published.

**Case:**

We report a completely asymptomatic mother of two FARAD children presenting exclusively with positive AChR antibodies. After birth, the first child needed intensive care therapy due to generalized hypotonia, respiratory problems, dysphagia, necessitating tube feeding and gastrostomy. FARAD was suspected because of ptosis, a hypomimic face, and confirmed by increased AChR antibodies in the mother. The mother became pregnant again 2 years later. Since FARAD is likely to reoccur and it is known that intensity of maternal myasthenia gravis treatment determines postnatal outcome, monthly intravenous immunoglobulin (IVIG) therapy was started at 12 weeks gestational age. The second child needed a short mask ventilation for initial stabilization at birth, but her muscle weakness improved rapidly and tube feeding was not necessary. Similar to her sister a tent-shaped mouth and a somewhat myopathic face persisted, but motor milestones were reached in time.

**Conclusions:**

These observations highlight that FARAD is an important differential diagnosis of genetically determined congenital neuromuscular disorders even in asymptomatic mothers, and that IVIG therapy during the pregnancy has the potential to improve the outcome of the children.

Fetal acetylcholine receptor antibody-associated disorders (FARAD) is a rare condition occurring in newborns of myasthenic mothers. The clinical spectrum ranges from a less severe predominantly myopathic phenotype, also known as fetal acetylcholine receptor inactivation syndrome (FARIS), to lethal arthrogryposis multiplex congenita (AMC) [1]. Until now, only two cases of an asymptomatic mother exclusively with positive acetylcholine receptor (AChR) antibodies giving birth to a FARAD child are described by Nguyen et al. (abstract only) [2] and Brueton et al. [3]. Subsequent pregnancies of mothers where MG had not been suspected can also result in AMC, even in more recently published cases [1]. Another third case describes a FARAD child born to an asymptomatic mother only showing elevated fetal AChR antibodies [4].

We present a novel, not yet published case of a FARAD patient whose mother was treated with intravenous immunoglobulin (IVIG) during pregnancy. The asymptomatic mother had no history for myasthenia gravis (MG) but showed elevated AChR antibodies. The diagnostic work-up was performed when her first child presented with typical signs of FARAD (see case 4.1 in the publication of Allen [1]. The older sister is the first child born at term by spontaneous vertex delivery without complications (Apgar 8/6/7, umbilical cord pH 7.18, birth weight 3340 g (P25-50), length 56 cm (P97), head circumference 37 cm (P97)). She had reduced respiratory efforts and was hypotonic, prompting mask ventilation and transport to the neonatal intensive care unit with continuous positive airway pressure (CPAP) ventilation. An X-ray suggested transient tachypnea of the newborn. However, there was also a right-sided high level of the diaphragm. CPAP therapy was continued for 3 days and feeding problems necessitated nasogastric tube feeding. Muscular hypotonia with a hypomimic face was noticed. Feeding problems slightly improved, but the girl still had to be fed by a nasogastric tube.

At the age of 3 months, the patient was admitted to our hospital for a second opinion. The girl had a hypomimic face and marked weakness of the head flexors and upper trunk muscles, contrasting with a normal muscle tone and a virtually normal strength of the lower extremities and the pelvic girdle. She had no ptosis, but a tent-shaped mouth. There were no jaw contractures or other contractures. The results of further diagnostics, including molecular genetic testing for myotonic dystrophy type 1 and disease-causing gene mutations concerning congenital myasthenic syndromes were normal. An electromyography and a cranial magnetic resonance imaging were also normal.

Serum creatine kinase level was regular (87 and 124 IU/l, normal: less than 146 IU/l). Repetitive nerve stimulation (RNS) of the ulnar nerve at a frequency of 3 Hz were regular. The determination of myasthenia-associated antibodies in the newborn’s blood serum (EUROIMMUN Medizinische Labordiagnostika AG, Lübeck, Germany) was negative.

Considering the clinical presentation of the child we assumed a clinically inapparent maternal MG. RNS was normal. Elevated AChR antibody immunoglobulin G (IgG) with 1.53 nmol/l (normal: less than 0.4 nmol/l) were detected, clinching the diagnosis of FARAD. Muscle-specific kinase (MuSK) antibodies were negative. The determination of maternal fetal AChR antibodies by radioimmunoassay or cell-based assay was not possible in our routine clinical diagnostics.

A percutaneous gastrostomy was performed at age 5 months due to persistent feeding problems. A salbutamol trial was started and increased up to 0.5 mg three times daily. This improved swallowing and reduced vomiting, but she still needed percutaneous endoscopic gastrostomy feeding until age 18 months. Her muscular hypotonia also improved. The child became able to sit at age 9 months and started free walking at age 20 months. She began to speak first words at age 18 months. Her voice was toneless due to velopharyngeal incompetence. A logopaedic therapy was initiated. At last follow-up at age 3 years, she was able to speak 3-word sentences, but her voice was still low and dysarthric. Chewing and swallowing were normal. Hearing was not impaired. Muscle strength and head control were also normal.

When the then 33-year-old was pregnant again, still with elevated AChR antibody IgG at 1.51 nmol/l, IVIG was given every 4 weeks at a dose of 1 g per kilogram of body weight starting at 12 weeks gestational age with the aim to decrease FARAD symptoms in the second child. The patient received a total seven infusions and IVIG was well tolerated. The mother remained stable without any myasthenic symptoms.

The second, also female child was born at 38 weeks of gestational age as spontaneous vaginal delivery (Apgar 1/4/8, umbilical cord pH 7.34, birth weight 2930 g (P10)) after induction of labor. Although fetal movements were normal, our neonatal intensive care unit team was present. Initially, a short mask ventilation was performed and oxygen was supplied for 4 h, but the newborn needed no further intensive care stay. The patient was hypomimic, slightly hypotonic, and showed some muscular weakness of the neck muscles. Head control was poor in the beginning, but feeding was uncomplicated. The baby was discharged home after 5 days. All clinical symptoms normalized during the first 2 months. AChR antibody IgG after birth were negative (0.2 nmol/l). The girl started turning around at age 4 months, sit without support at age 7 months, walked without support at age 14 months, and spoke first words at age 12 months. She was followed up until age 18 months. Overall, her symptoms were much less severe compared to the first-born sister.

Unlike transient neonatal myasthenia gravis persisting 4–6 weeks, FARAD is a myopathy which extends well beyond the neonatal period [2]. It is characterized by an early-onset ingrained endplate myopathy associated with maternal AChR antibodies directed against the fetal γ subunit of the nicotinic acetylcholine receptor [5]. Therefore, the lack of adult AChR antibodies in our FARAD children is not unexpected. The typical phenotype, along with persistent paralysis of facial muscles, a tent-shaped mouth and speech difficulties together with the elevated AChR antibodies in the mother lead to the diagnosis of FARAD.

Mothers with a previously affected child have a high reoccurrence rate in future pregnancies [6, 7]. IVIG have been used in pregnancy to treat autoimmune diseases and are considered safe. Pharmacovigilance studies have not reported any malformations associated with the intravenous administration of immunoglobulins [8]. Various mechanisms for IVIG activity have been hypothesized including competition with AChR antibody binding to AChR [9]. Gamez and co-workers reported on a successful monthly IVIG treatment of five pregnant MG women beginning two months before planning pregnancy. Infusions were well tolerated and the newborns were healthy [8]. Regarding the side effects, invasiveness of other therapies like plasma exchange as well as the asymptomatic mothers’ clinical status, IVIG treatment was chosen. Regarding the beginning of the placental transfer of IgG at 14–16 weeks [5] we initiated our reported IVIG therapy regime at week 12 of gestational age expecting a reduction of maternal autoantibodies. The pregnancy immunotherapy treatment score proposed by Allen [1], evaluating mode and timing of immunotherapy, is 7.5 for our mother. Beside the timing and intensity of treatment, other possible factors for the varying disease severity are the level of antibodies to fetal AChR and timing of conversion of fetal to adult AChR in different muscle groups [10]. Compared to the higher mean score of 12.9 associated with less severely affected offspring presented by Allen [1] earlier treatment could have had a positive impact on our newborn.

Initial clinical status of the second-born was life-threatening but IVIG treatment of the mother resulted in a positive impact on the following clinical course of the child.

In summary, our case demonstrates that diagnosing FARAD is notably challenging when the mother does not suffer from MG but shows exclusively elevated AChR antibodies. IVIG treatment seems to have a positive impact on the clinical severity of FARAD highlighting its important clinical implications for future pregnancies, therapeutic options and the postnatal outcome.

## Data Availability

Not applicable.

## Abbreviations

AChR: Acetylcholine receptor
APGAR: appearance, pulse, grimace, activity, respiration
AMC: arthrogryposis multiplex congenita
CPAP: continuous positive airway pressure
FARAD: fetal acetylcholine receptor antibody-associated disorders
FARIS: fetal acetylcholine receptor inactivation syndrome
IgG: immunoglobulin G
IVIG: intravenous immunoglobulin
MuSK: muscle-specific kinase
MG: myasthenia gravis
RNS: repetitive nerve stimulation
